# Aging: Cancer – an unlikely couple

**DOI:** 10.18632/aging.101295

**Published:** 2017-09-20

**Authors:** Antja-Voy Hartley, Matthew Martin, Tao Lu

**Affiliations:** Department of Pharmacology and Toxicology, Indiana University School of Medicine, Indianapolis, IN 46202, USA

**Keywords:** aging, cancer, epigenetics, inflammation, senescence

At first glance, the molecular and cellular mechanisms governing aging and cancer appear to be completely distinct, even opposite in terms of their phenotypes. On one hand, aging represents a slow, often degenerative decline in cellular functions, while cancer cells are thought be “hyper-functional”. But are they really that opposite? It is no secret that aging increases one's susceptibility to many diseases, including cancer. In fact, as aging populations throughout the world increase, there has been an unprecedented rise in cancer incidence and mortality. More than 50 percent of all cancers are diagnosed in patients 65 years or older [1]. There is now more than ever an urgent need to better understand the interplay between aging and cancer. This knowledge will undoubtedly improve clinical management of geriatric cancer patients. Here, we discuss some intriguing factors linking these two seemingly paradoxical conditions.

There are several age-related epigenetic defects reminiscent of those commonly observed in cancer, suggesting a potential link between age-dependent biological changes and the concomitant increased cancer risk observed in the aged population. These include several global changes in the chromatin, altered gene expression, and overall genomic instability. For instance, certain site-specific hypermethylation events at promoters of genes involved in aging have also been reported as occurring during cancer. Notably, these include hypermethylation patterns at tumor-suppressor genes, such as suppressor of cytokine signaling 1 (*SOCS1*), hypermethylated in cancer 1 (*HIC1*), adenomatosis polyposis coli (*APC*), and a set of genes collectively termed the Polycomb group protein (PcG) target genes [2]. The resulting epigenetic silencing of these genes typically precedes many of the transformation events that give rise to cancer, further underscoring the idea that elucidating changes in the DNA methylome during aging may provide a powerful platform for better understanding the factors contributing to tumorigenesis.

Aged-related changes in genomic imprinting constitute another aspect of epigenetic alterations in cancer. For instance, loss of imprinting (LOI) for the insulin growth factor 2 (IGF2) has been shown to be important in cancer progression. Fu *et al* demonstrated that increased IGF2 expression occurred as a result of selective LOI for IGF2 in the prostate of men during aging which was shown to be more extensive in men with age-associated prostate cancer [3].

In addition to epigenetic changes, senescence also stands out as an important link between aging and cancer. First described by Hayflick and Moorhead in 1961 [4], age-associated senescence denotes a cell-autonomous state in which cells undergo irreversible cell cycle arrest marked by certain “Hayflick factors”, such as telomere loss, genomic instability, oxidative stress, and the accumulation of DNA damage [4]. Interestingly, many of these factors are intrinsic components of common age-related types of cancer, including breast, skin, and lung cancers [5].

Although senescence has been recognized to be a tumor suppressor mechanism due to its strong antiproliferative power, ironically, emerging evidence suggests that as the number of senescent cells increase with age, a more permissive tissue microenvironment develops which may, contribute significantly both to the initiation and progression of cancer. This is facilitated by a process that develops in advanced senescent cells known as the senescence-associated secretory phenotype (SASP) [6]. SASP is characterized by senescence-associated changes in which robust levels of cytokines, chemo-kines, and growth factors secreted into the surrounding microenvironment, acting on premalignant and tumor cells in a paracrine fashion, promoting a highly inflammatory state that enhances the invasive capabilities of these cells. Not surprisingly, this parallels a major pervasive feature of aging, *i.e.* a persistent state of chronic and systemic inflammation, which has been shown to exponentially increase the risk of cancer development. SASP is largely initiated by interleukin 1 alpha and beta (IL-1α and β), which serve as potent activators of important cancer signals, including the nuclear factor-κB (NF-κB) and p38 mitogen-activated protein kinase (p38MAPK) pathways. These pathways along with SASP can then serve to further activate a wide array of critical target gene products including those involved in epithelial-to-mesenchyme transition (EMT) (*eg.* Metallomatrix proteins (MMPs), and metastasis (*eg*. MMPs, IL-6) of cancer cells [6].

**Figure 1:**
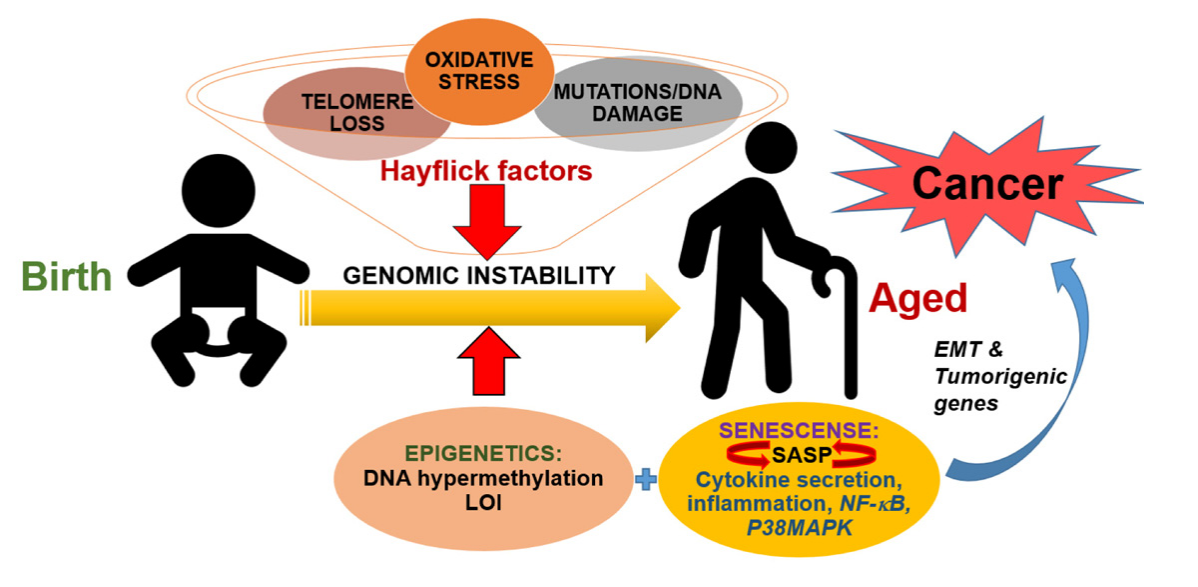
Mechanisms of interplay between aging and cancer During normal aging, accumulation of damaged DNA, telomere loss, mutations and subsequent oxidative stress-dependent changes, the so-called “Hayflick factors” lead to overall genomic instability. Meanwhile, the increasing abundance of aberrant epigenetic changes such as DNA hypermethylation and loss of imprinting (LOI) can also significantly contribute to genomic instability. Yet another complication arises as these advanced senescent cells develop the senescence-associated secretory phenotype (SASP), which is accompanied by robust cytokines secretion that can act on premalignant cells to promote a highly inflammatory tissue microenvironment. SASP itself is initiated by certain pro-inflammatory cytokines which serve as potent activators of important inflammatory signals, including the nuclear factor-κB (NF-κB) and p38 mitogen-activated protein kinase (p38MAPK) pathways. These pathways, once activated, can also establish a feed-forward loop via the transcription of cytokine target genes that in turn activate SASP. Overall, this results in activation of an epithelial-to-mesenchyme transition (EMT) and tumorigenic phenotype in cells, due to increased transcription of gene products related to these processes. This ultimately leads to cancer initiation and progression.

Therapeutic strategies for treating elderly cancer patients are influenced by several complicating factors, many of which stem from natural frailty and other age-related comorbidities that disproportionately affect this population. For instance, severity of age-related ailments, impaired renal/hepatic function, and altered metabolism of therapeutics are just a few factors that influence tolerance for the toxicity associated with more aggressive chemotherapeutic and radiation regimens [5]. Furthermore, this is compounded by an alarming underrepresentation of older patients in randomized cancer-related trials, leading to a paucity of evidence-based benefit of newer, more effective drugs in older cancer patients [7]. By developing more appropriate clinical trials dedicated to older individuals, the hope is that further elucidation of the interplay between aging and cancer will give clinicians much-needed improve-ment in risk assessment and therapeutic intervention strategies to improve survival and quality of life for these patients (Figure 1).
